# Novel techniques in the prophylaxis and treatment of type II endoleaks: a narrative review

**DOI:** 10.3389/fcvm.2026.1847190

**Published:** 2026-10-19

**Authors:** Krzysztof Pietrzak, Maciej Koperski, Klaudia Gutowska, Piotr Terlecki

**Affiliations:** 1 Department of General and Vascular Surgery, Provincial Hospital in Bielsko-Biała, Bielsko-Biala, Poland; 2 Medical University of Lodz, Łódź, Poland; 3 Department of Obstetrics and Gynecology, Institute of Mother and Child, Warsaw, Poland; 4 Department and Clinic of Vascular Surgery and Angiology, Medical University of Lublin, Lublin, Poland

**Keywords:** abdominal aorta aneurysm, aneurysm, embolization, endovascular aneurysm repair, type II endoleaks

## Abstract

**Background:**

Type II endoleaks (T2ELs) are a frequent complication after endovascular aneurysm repair (EVAR), posing a risk of aneurysm expansion. This review evaluates modern techniques for T2ELs management, moving beyond traditional embolization methods.

**Methods:**

Recent controlled trials and observational studies were analyzed to trace evidence on conventional and novel techniques employed in managing T2ELs.

**Results:**

Novel techniques, such as transcaval embolization (TCE) and laser-assisted transgraft embolization (LATE), have demonstrated encouraging early technical and clinical outcomes, although the available evidence remains limited. In addition, shape memory polymer (SMP) embolization resulted in significant sac shrinkage in >85% of patients and prevented clinically significant T2ELs.

**Conclusions:**

TCE, LATE, and prophylactic SMP embolization are promising alternatives to conventional methods, offering high technical success and the potential to reduce reinterventions. Larger studies are needed to confirm long-term outcomes and define optimal use.

## 1 Introduction

Endovascular aneurysm repair (EVAR) has become the preferred treatment modality for abdominal aortic aneurysms (AAA), offering reduced perioperative morbidity and mortality compared to open surgery (1). However, its long-term durability can be compromised by endoleaks, the most common complication following EVAR, occurring in up to 30% of patients (2). Among these, Type II endoleaks (T2ELs)—caused by retrograde flow from collateral vessels such as the inferior mesenteric or lumbar arteries—account for approximately 10%–20% of cases (3).

Although often considered benign, persistent T2ELs may lead to aneurysm sac expansion, rupture, or the need for secondary interventions (4). Consequently, management of T2ELs remains a debated topic, with treatment options evolving from conservative surveillance to increasingly sophisticated endovascular interventions.

Traditional techniques, including transarterial and translumbar embolization, have shown variable success rates. Transarterial embolization is limited by anatomical challenges and failure to access feeding vessels (5), while direct sac puncture via translumbar access carries procedural risks and requires precise targeting (6).

In recent years, innovative strategies have emerged to improve both access and efficacy in T2EL treatment. These include transcaval embolization, in which the aneurysm sac is accessed via the inferior vena cava using specialized imaging guidance (7), laser-assisted transgraft embolization, which utilizes laser fenestration of the endograft fabric to deliver coils directly into the sac (8) and prophylactic aneurysm sac embolization during the primary EVAR procedure using materials such as shape memory polymers (SMPs) to prevent T2ELs before they develop (9).

These approaches aim to address the limitations of conventional methods by improving technical feasibility and long-term outcomes. Yet, despite growing interest, the relative efficacy, safety, and indications for these emerging techniques remain incompletely defined.

The aim of this review is to provide a comprehensive overview of contemporary methods for the management of Type II endoleaks, with particular emphasis on these novel approaches, their underlying rationale, procedural techniques, and clinical outcomes. By integrating current evidence, we seek to guide vascular specialists in tailoring endoleak management strategies to individual patient anatomy and risk profiles.

## 2 Materials and methods

The literature search was performed using PubMed, Embase, Science Direct and Scopus databases from inception until August 2025. Keywords included: “Type II endoleaks”, “T2ELs”, “Endovascular aneurysm repair”, “EVAR”, AND with “complication”, “embolisation”, “management”, “TCE”, “LATE”, “SMP”, “TAE”. Two reviewers (K.G. and K.P.) independently screened titles and abstracts for relevance. Discrepancies were resolved through discussion and consensus. Eligible study types included Polish and English-language articles—controlled trials and observational studies were eligible. Studies with missing data, not adressing research question were exluded. Priority was given to most recently published studies. For the purposes of this review, technical success was defined as successful access to the target site and completion of the intended embolization procedure. Clinical success was defined as the absence of clinically relevant aneurysm sac expansion during follow-up, with no need for T2EL-related reintervention. However, definitions of technical and clinical success varied among the included studies; therefore, study-specific definitions were retained when reporting individual study outcomes.

## 3 Conventional techniques

Management of type II endoleaks has traditionally followed a conservative-first approach, with treatment reserved for cases of persistent endoleak associated with aneurysm sac expansion (typically >5 mm over 6–12 months) (10, 11). Once intervention is indicated, the two most established endovascular techniques are transarterial embolization and direct sac puncture, most often via the translumbar approach.

### 3.1 Imaging evaluation and surveillance

Accurate diagnosis and follow-up of T2ELs depend heavily on imaging. Computed tomography angiography (CTA) remains the gold standard for detection and surveillance, offering excellent spatial resolution and allowing evaluation of sac morphology, endoleak size, and collateral vessels. However, CTA may overestimate the presence of persistent T2ELs due to delayed venous filling or flow artifacts (12).

Contrast-enhanced ultrasound (CEUS) has gained interest due to its ability to identify active flow in low-pressure endoleaks with high sensitivity and without the need for nephrotoxic contrast or radiation exposure. Studies have shown CEUS to outperform CTA in the detection of persistent or recurrent T2ELs in selected patients (13). In clinical practice, both modalities are often used complementarily, especially in cases of equivocal findings.

### 3.2 Transarterial embolization

Transarterial embolization (TAE) involves selective catheterization of the feeding artery, most commonly the inferior mesenteric artery (IMA) or lumbar arteries, via the superior mesenteric artery, internal iliac arteries, or other collaterals. Embolic materials include coils, glue, Onyx, or gelfoam (14). TAE is typically performed under fluoroscopic guidance and may be preceded by CTA-based mapping of the collateral pathways.

Recent advances in imaging have refined the performance of TAE. In particular, the use of cone-beam CT with three-dimensional image fusion allows more precise visualization of the nidus and collateral pathways during embolization. Zaarour et al. reported in a series of 37 patients that fusion-guided TAE or direct sac puncture achieved 95% immediate technical success and 88% success at 1 year, with significantly reduced procedure time and contrast use compared with conventional guidance (15).

While TAE is minimally invasive and familiar to most interventionalists, its effectiveness is limited by technical challenges, especially in accessing small, tortuous feeders. Reported technical success rates vary widely, from 50% to 90%, and recurrence rates remain high, with up to 30% of patients requiring repeat interventions (16). Non-target embolization and spinal cord ischemia, although rare, are recognized risks.

### 3.3 Direct sac puncture (translumbar approach)

In cases where transarterial access fails or is not feasible, direct sac puncture via a translumbar route provides an alternative. Under CT or fluoroscopic guidance, a spinal needle is advanced into the aneurysm sac, followed by embolization with coils, thrombin, glue, or Onyx (17). This approach allows direct treatment of the endoleak cavity and can be more effective when a specific feeding vessel is not identifiable.

However, translumbar embolization carries procedural risks, including retroperitoneal hemorrhage, injury to surrounding organs, and patient discomfort due to the prone position. Moreover, the success of sac puncture is highly dependent on anatomical factors and operator expertise. In comparative studies, translumbar access has demonstrated higher technical success and lower recurrence rates compared to TAE, albeit with slightly increased procedural complexity (6).

### 3.4 Limitations of conventional techniques

Despite their widespread use, both TAE and translumbar embolization are limited by incomplete sac exclusion, recanalization, and technical failure in complex anatomies. Additionally, neither approach directly addresses the underlying risk of recurrent collateral flow, which may explain the high rates of persistent T2EL despite technically successful embolization. These limitations have spurred interest in alternative approaches, including transcaval access, intrasac embolization via laser fenestration, and preemptive embolization strategies.

## 4 Transcaval embolization

Transcaval embolization (TCE) has emerged as an innovative and increasingly adopted approach to treating type II endoleaks, especially in patients with complex aortic anatomies or failed prior interventions. This technique exploits the anatomic proximity of the inferior vena cava (IVC) to the abdominal aortic aneurysm sac, offering a direct yet minimally invasive route for endoleak embolization. Originally developed as a salvage option, TCE is now being used electively in select centers due to its high technical success rates and low morbidity.

The procedure involves percutaneous femoral venous access followed by transvenous catheter navigation into the IVC. Using fluoroscopic or cone-beam CT guidance, the aneurysm sac is then accessed through the cava with either a mechanical needle or, more recently, a chronic total occlusion (CTO) guidewire electrified with electrocautery to create a controlled fenestration (18). Once access to the sac is confirmed, embolic agents including detachable coils, Onyx, thrombin, or shape memory materials are deployed to occlude the flow channel and promote thrombosis.

Several refinements have improved the safety and efficacy of this technique. In particular, the use of intravascular ultrasound (IVUS) or intracardiac echocardiography (ICE) allows real-time imaging of the aortic wall and sac, enhancing puncture accuracy and reducing complications (19). Furthermore, steerable sheaths and low-profile delivery systems enable more precise catheterization of target vessels within the sac, although selective embolization of outflow vessels remains technically demanding and often incomplete.

Clinical outcomes of transcaval embolization have been encouraging. In a 2022 systematic review including 117 patients across 8 studies, the pooled technical success rate was 91.4%, with a persistent endoleak rate of only 12.8% during a mean follow-up of 12 months (7). Procedure-related complications were rare, and reintervention rates were comparable or lower than those seen with translumbar or transarterial techniques. Individual institutional series have also reported promising results: Patel et al. described a 100% access success rate using an electrified CTO wire and liquid embolics, with only one minor bleeding event and no procedural mortality (19).

Despite these benefits, transcaval embolization is not without limitations. Steerability of the catheter across the IVC-aorta interface remains a challenge, often preventing complete outflow vessel embolization. In the study by Patel et al., selective cannulation of outflow vessels was successful in only 16% of cases (19). Moreover, while access-related complications are infrequent, inadvertent damage to the aortic endograft or adjacent structures remains a theoretical risk, particularly in heavily calcified vessels. Rare but serious complications such as aortic-caval fistula formation or sac infection have also been reported, albeit infrequently (19).

Patient selection is a key determinant of success. Ideal candidates include those with anatomically favorable proximity of the IVC to the aneurysm sac, prior failed transarterial/translumbar interventions, or significant comorbidities that preclude direct sac access under general anesthesia. Notably, TCE has also been used in the context of failed sac regression after EVAR without overt endoleak, where occult type II leaks are suspected.

In summary, transcaval embolization represents a valuable and technically feasible alternative for managing type II endoleaks. Available evidence suggests that TCE may represent a technically feasible alternative for managing type II endoleaks, particularly when traditional access routes are not viable. Further studies are required to better define its safety, long-term efficacy, and role in treatment algorithms.

## 5 Transgraft embolization

### 5.1 Laser-assisted techniques

Laser assisted transgraft embolization (LATE) is a technically innovative method developed to overcome the limitations of traditional access routes for treating type II endoleaks (T2ELs), particularly in cases where translumbar, transarterial, or transcaval approaches are not feasible or have previously failed. The technique enables direct catheterization of the aneurysm sac through the fabric of the stent graft using an endovascular excimer laser.

The procedure typically involves advancing a steerable sheath (e.g., TourGuide, Medtronic) through a femoral arterial approach. A 2.3-mm excimer laser catheter (Turbo-Elite, Philips) is then used to perforate the graft fabric at a preselected location usually guided by overlay fusion imaging or cone-beam computed tomography. Once the sac is accessed, target vessels are selectively embolized using coils or liquid embolics. Crucially, the laser fenestration is re-lined using a covered stent or cuff to seal the iatrogenic defect and prevent type IIIb endoleak formation (8, 20).

Mehta et al. recently described the largest clinical series to date, reporting on five patients with eleven target vessels embolized via laser-assisted transgraft access (8). The technical success rate was 100%, with no major perioperative complications. All patients demonstrated either stability or regression of aneurysm sac diameter on follow-up imaging. The authors emphasized the necessity of post-fenestration relining and suggested that this technique may offer a safe and effective solution in anatomically complex cases.

Other reports have supported its feasibility and reproducibility. Mewissen et al. first described the method in 2017, reporting successful coil embolization through laser-created fenestrations in three patients (21). Boehme et al. extended the experience by applying this approach to T2ELs located near graft limbs and accessory renal arteries (22). Kuwayama published a detailed “How I Do It” procedural guide highlighting the importance of pre-procedural planning, graft material compatibility, and relining strategies (20).

This approach offers several advantages. First, it provides direct and coaxial access to the aneurysm sac, allowing easier catheter manipulation and improved control during embolization. Second, unlike transcaval or translumbar routes, it avoids crossing retroperitoneal tissue planes or the vena cava. Third, it can be performed entirely endovascularly, without general anesthesia in many cases.

However, several limitations must be considered. The requirement for specialized laser equipment and operator expertise may limit accessibility. Moreover, while no type III endoleaks have yet been observed in the published series, the potential for endograft damage remains a concern. Relining is essential to mitigate this risk. Long-term durability data are lacking, and the cumulative cost of laser catheters and additional graft material may be significant.

In clinical practice, laser-assisted transgraft embolization is currently best reserved for highly selected patients, particularly those with anteriorly located or graft-adjacent endoleaks and those with prior failed embolization attempts. As experience accumulates, this technique may represent an adjunctive option in centers with appropriate endovascular expertise.

### 5.2 Long-needle techniques

Transgraft embolization using a long custom needle (LCN) represents another innovative method to access the aneurysm sac when conventional routes are not feasible. Instead of laser fenestration, this approach relies on direct puncture of the stent graft fabric under fluoroscopic and fusion imaging guidance, allowing catheterization of the sac without the need for specialized laser equipment.

Matsumura et al. reported the first dedicated case series of long-needle transgraft embolization in 10 patients with persistent type II endoleaks after EVAR (23). Technical success was achieved in all cases, with target vessels embolized using coils and/or liquid embolics. At a mean follow-up of 24 months, nine patients demonstrated stable or regressing aneurysm sacs, and only one required repeat intervention for recurrent endoleak. Importantly, no type III endoleaks or major perioperative complications were observed.

The long-needle technique offers several practical advantages. It is less resource-intensive than laser-assisted approaches, since it does not require excimer laser equipment, and may be performed with standard interventional radiology tools modified for vascular puncture. It provides direct and controlled access to the nidus, potentially improving embolization efficacy in anatomically challenging cases. However, the main limitation is the need for a custom extra-long needle, which is not widely available, and concerns remain about long-term durability given the limited sample size.

Overall, early results suggest that the long-needle method is a technically feasible adjunct in the management of type II endoleaks, particularly in patients unsuitable for translumbar, transcaval, or laser-assisted transgraft embolization. Further studies with larger cohorts and longer follow-up are needed to establish its safety, clinical efficacy, and durability.

## 6 Prophylactic sac embolization with shape memory polymer (SMP)

Prophylactic embolization of the aneurysm sac at the time of EVAR represents a paradigm shift in the management of type II endoleaks (T2ELs). Rather than treating endoleaks after they occur, this technique aims to prevent their development altogether by filling the sac with embolic material during the initial EVAR procedure. Among the most promising tools for this purpose are shape memory polymer (SMP) plugs, which offer a novel biomaterial-based approach to sac thrombosis.

Shape memory polymers are porous, self-expanding materials that change shape upon contact with blood or body temperature. The most widely studied device to date is the IMPEDE-FX embolization plug (Shape Memory Medical, Inc.), which is delivered through standard 0.035-inch catheters. Once deployed into the sac, the polymer expands slowly, conforming to the aneurysm cavity and creating a scaffold that promotes thrombus formation without relying on coils or liquid agents (9).

Early clinical experience with SMP-based sac embolization has been encouraging. In a multicenter prospective study by Massmann et al., 35 patients underwent EVAR with concurrent sac embolization using shape memory polymer (9). The technical success rate was 100%, with no device-related complications or systemic adverse events. During a median follow-up of 11 months, significant sac shrinkage was observed in over 85% of patients, and notably, no secondary interventions were required for T2ELs. Importantly, even in cases where Type II endoleaks were detected on imaging, these did not lead to sac growth, suggesting that the SMP-induced thrombosis mitigated their clinical significance. O'Donnell et al. reported outcomes of 29 patients treated with SMP plugs during infrarenal EVAR across two centers, with one-year follow-up available for 22 patients (24). The authors demonstrated no perioperative complications, no endoleaks, and no reinterventions, while the majority of patients achieved aneurysm sac regression by both volume (67% with >10% reduction) and diameter (55% regression, 45% stability). Importantly, SMP plugs are biodegradable and do not produce imaging artifacts, which may provide an advantage over coils or liquid embolics in postoperative surveillance.

More recently, Mukherjee et al. reported favorable 1-year results with preemptive SMP sac embolization combined with a conformable neck sealing endograft, further supporting the feasibility of this approach in contemporary EVAR practice (25).

The SMP plug offers several theoretical advantages over traditional embolic agents. Its slow, controlled expansion minimizes the risk of non-target embolization. The porous structure encourages host tissue in-growth, which may enhance long-term stability and integration. Unlike liquid agents or coils, SMP devices do not require precise delivery into specific feeding vessels, which simplifies the procedure and reduces dependence on catheter steerability (9). In contrast to gelfoam or thrombin, SMP does not degrade or resorb, suggesting more durable sac exclusion.

Preclinical and early clinical studies have also demonstrated biocompatibility, low inflammatory response, and no imaging artifact, which facilitates post-procedural CTA interpretation (26). Furthermore, because the device can be deployed via a contralateral limb sheath or dedicated catheter, it adds minimal time or complexity to the primary EVAR procedure.

However, several limitations remain. The technique is currently off-label in many regions and not yet widely reimbursed. Long-term outcome data beyond 1–2 years are lacking, and comparative studies with coil-based or glue-based prophylactic embolization have not yet been published. Additionally, deployment requires careful preoperative planning and familiarity with sac morphology to avoid malposition or excessive filling pressure.

Despite these caveats, shape memory polymer-based sac embolization has shown strong early promise as a preventive strategy for T2ELs. It may be especially beneficial in high-risk anatomies, such as large aneurysm sacs with multiple patent lumbar arteries or prominent inferior mesenteric branches, where traditional endoleak treatment is more likely to fail. Further prospective studies are needed to determine whether these encouraging early findings translate into reduced reintervention rates and improved long-term outcomes (Table 1).

**Table 1:** Comparative overview of conventional and emerging techniques for prevention and management of type II endoleaks after EVAR.

Technique	Key evidence	Typical indications	Technical aspects	Reported outcomes	Potential strengths	Current limitations
Transarterial embolization (TAE)	Multiple retrospective comparative studies [Stavropoulos et al. (5); Yang et al. (6); Zaarour et al. (15)]	Patent IMA or lumbar artery Favorable collateral anatomy First-line endovascular treatment	Transarterial catheterization of feeding artery Selective nidus embolization Coils and/or liquid embolics	Variable clinical success Recurrence not uncommon Repeat embolization frequently required	Widely avaible Established technique Well-documented outcomes	Challenging collateral catheterization Lower efficacy with complex anatomy Variable clinical success and relatively common recurrence
Direct translumbar sac puncture	Multiple retrospective comparative studies [Yang et al. (6); Zener et al. (17)]	Failed or unsuitable TAE Direct percutaneous access feasible Complex collateral anatomy	CT- or fluoroscopy-guided sac puncture Direct nidus embolization Liquid embolics and/or coils	High technical success Good sac stabilization Low recurrence in contemporary series compared to TAE	Direct access to endoleak cavity Effective when the feeding vessel is not identifiable Well-documented outcomes	Safe access route required Not feasible in all patients Access-site complications
Transcaval embolization (TCE)	Systematic review and retrospective series [Nana et al. (7); Van Sickler et al. (18); Patel and Miller (19)]	Failed conventional embolization No safe posterior access Unfavorable arterial anatomy Suitable anatomy for transcaval access	Femoral venous access Caval wall puncture into aneurysm sac Sac feeder embolization with coils, Onyx, thrombin or shape memory materials	High technical success Sac stabilization in most reports Persistent T2EL may occur	Alternative access route Avoids difficult collateral navigation	Retrospective evidence only Technique heterogeneity Limited long-term outcome data
Laser-assisted transgraft embolization (LATE)	Case reports and small case series [Mewissen et al. (21); Kuwayama (20); Mehta et al. (8)]	Failed conventional embolization Anterior endoleaks Need for selective feeder catheterization	Arterial access and laser graft fenestration Direct endograft-to-sac access Selective feeder embolization followed by graft relining	High technical success rate Favorable clinical outcomes reported	Direct and coaxial access to the aneurysm sac Avoids retroperitoneal access Entirely endovascular performance	Limited efficacy evidence and no long-term follow-up data Specialized equipment and high cost Mandatory graft relining
Long-needle transgraft embolization	Single-center case series [Matsumura et al. (23)]	Failed or unsuitable conventional embolization Persistent T2EL	Arterial access and needle puncture through graft fabric Direct sac access Nidus embolization	High technical success Sac regression reported	No laser required Standard endovascular equipment Direct nidus access	Limited efficacy evidence and no long-term follow-up data
Prophylactic shape memory polymer (SMP) sac embolization	Early clinical case series [Massmann et al. (9); O'Donnell et al. (24); Mukherjee et al. (25)]	High predicted risk of T2EL	Intra-sac SMP deployment during EVAR	High technical success No secondary interventions for T2EL needed	Preventive approach Radiolucent materials	Limited efficacy evidence and no long-term follow-up data

## 7 Discussion

The optimal management of T2ELs remains controversial, primarily because their natural history is heterogeneous and the risk associated with the endoleak itself appears to be substantially lower than that associated with progressive aneurysm sac expansion. This is reflected in contemporary guidelines, which generally support surveillance rather than routine intervention in patients with T2ELs and stable sac diameter. The Society for Vascular Surgery (SVS) guidelines recommend surveillance of T2ELs without aneurysm expansion and suggest treatment when sac expansion occurs; an increase of ≥5 mm is considered clinically relevant in the accompanying discussion (27). More recently, the 2024 European Society for Vascular Surgery (ESVS) guidelines adopted a more conservative threshold, recommending that secondary intervention should be considered when sac growth reaches ≥10 mm compared with baseline or the smallest diameter recorded during follow-up, provided that alternative causes of sac expansion, particularly type I or III endoleaks, have been excluded (28). Although the precise intervention threshold therefore differs between the two guidelines, both emphasize sac enlargement rather than the presence of T2EL alone as the principal indication for treatment.

This conservative approach is broadly consistent with the evidence reviewed in this article. T2ELs frequently resolve or remain clinically stable, whereas rupture associated with isolated T2EL is uncommon (2). Conversely, progressive sac enlargement represents a clinically relevant adverse finding after EVAR and provides a more meaningful therapeutic target than technical elimination of endoleak flow alone. Importantly, successful embolization does not invariably translate into durable sac stabilization. Long-term follow-up has demonstrated that recurrent or persistent sac expansion and the need for repeat intervention remain substantial despite initially successful embolization (16). These observations also support the distinction between technical and clinical success and highlight the importance of continued imaging surveillance after intervention.

From a clinical perspective, these data support a stepwise management strategy. T2EL without significant sac enlargement should generally remain under imaging surveillance. In patients with progressive sac expansion, occult type I or III endoleaks should first be excluded before attributing aneurysm growth to T2EL. When intervention is indicated, an endovascular approach should be preferred, with the access route selected according to endoleak anatomy, previous interventions, and local expertise. Transarterial embolization and direct sac puncture remain the most established approaches, while transcaval or transgraft access may provide alternatives when conventional routes are technically difficult or have previously failed. Persistent aneurysm growth despite endovascular treatment should prompt reassessment of the endoleak etiology and, in selected surgically fit patients, consideration of open conversion, as also reflected in the ESVS 2024 recommendations (28).

The emerging techniques discussed in this review may gradually modify this treatment paradigm, but the current evidence does not justify their routine use. Transcaval embolization has accumulated encouraging evidence, including high technical success in systematic review data, but available studies remain heterogeneous and predominantly observational (7). Evidence for transgraft techniques is even more limited and currently supports their use mainly as alternative access strategies in selected anatomically challenging cases. Similarly, prophylactic embolization represents a potentially important future direction, particularly if patients at high risk of persistent T2EL and subsequent sac expansion can be reliably identified. However, the ESVS 2024 guidelines currently recommend against routine pre-emptive embolization of the inferior mesenteric artery, lumbar arteries, or non-selective aneurysm sac embolization because a clinically relevant long-term benefit has not yet been sufficiently demonstrated (28). Consequently, future changes in clinical recommendations will require prospective studies demonstrating not only technical prevention or elimination of T2EL, but also durable reduction in sac expansion, reintervention, and aneurysm-related complications. Until such evidence becomes available, treatment should remain selective and primarily driven by aneurysm sac behavior rather than the presence of T2EL alone.

## 8 Conclusions

Type II endoleaks remain a common challenge following EVAR, particularly in anatomically complex patients or those with persistent sac expansion. While traditional approaches such as transarterial and translumbar embolization remain the standard treatment strategies, emerging techniques—including transcaval embolization, laser-assisted transgraft embolization, and prophylactic sac embolization using shape memory polymers—have demonstrated encouraging early technical and clinical results. These methods expand the armamentarium for endoleak management and may provide effective alternatives in selected clinical scenarios. However, the currently available evidence is largely based on retrospective studies, case series, and small patient cohorts. Direct comparisons between techniques remain scarce, outcome definitions are heterogeneous, and long-term durability has not yet been established. Continued evaluation through larger prospective studies is essential to define the long-term efficacy and safety of these novel methods and to establish evidence-based criteria for selecting the most appropriate treatment approach for individual patients.
